# A Novel Thermochemical Metal Halide Treatment for High-Performance Sb_2_Se_3_ Photocathodes

**DOI:** 10.3390/nano11010052

**Published:** 2020-12-28

**Authors:** Svetlana Polivtseva, Joseph Olanrewaju Adegite, Julia Kois, Damir Mamedov, Smagul Zh. Karazhanov, Jelena Maricheva, Olga Volobujeva

**Affiliations:** 1 Department of Materials and Environmental Technology, TalTech, School of Engineering, Ehitajate tee 5, 19086 Tallinn, Estonia; jelena.maricheva@taltech.ee (J.M.); olga.volobujeva@taltech.ee (O.V.); 2 Mechanical Engineering Department, Worcester Polytechnic Institute, 100 Institute Road, Worcester, MA 01609, USA; joadegite@wpi.edu; 3 LLC Auramet, Kalliomäentie 1B, 02920 Espoo, Finland; juliakois2@gmail.com; 4 Department of Materials Science, National Research Nuclear University (MEPhI), 115409 Moscow, Russia; damir.mamedov@ife.no (D.M.); smagulk@ife.no (S.Z.K.); 5 Department for Solar Energy, Institute for Energy Technology, NO-2027 Kjeller, Norway

**Keywords:** Sb_2_Se_3_, chemical post-deposition treatment, annealing, chemical activation, high-performance photocathode

## Abstract

The fabrication of cost-effective photostable materials with optoelectronic properties suitable for commercial photoelectrochemical (PEC) water splitting represents a complex task. Herein, we present a simple route to produce Sb_2_Se_3_ that meets most of the requirements for high-performance photocathodes. Annealing of Sb_2_Se_3_ layers in a selenium-containing atmosphere persists as a necessary step for improving device parameters; however, it could complicate industrial processability. To develop a safe and scalable alternative to the selenium physical post-processing, we propose a novel SbCl_3_/glycerol-based thermochemical treatment for controlling anisotropy, a severe problem for Sb_2_Se_3_. Our procedure makes it possible to selectively etch antimony-rich oxyselenide presented in Sb_2_Se_3_, to obtain high-quality compact thin films with a favorable morphology, stoichiometric composition, and crystallographic orientation. The treated Sb_2_Se_3_ photoelectrode demonstrates a record photocurrent density of about 31 mA cm^−2^ at −248 mV against the calomel electrode and can thus offer a breakthrough option for industrial solar fuel fabrication.

## 1. Introduction

Continuously increased global energy consumption provokes the development of substitutive solutions such as thin-film solar (TFSCs) [1,2,3] or PEC water splitting cells [4]. To follow the strategy toward a green society, solutions should simultaneously satisfy high efficiency and cost-effectiveness criteria. Inorganic TFSCs have recently demonstrated remarkable power conversion efficiencies (PCE): 23.35% for the copper-indium-gallium-selenide (CIGS) technology in 2019 [2], or 22% for cadmium telluride (CdTe) in 2017 [3]; and the PEC cells exploiting III–V photovoltaic materials reached a solar-to-hydrogen (STH) efficiency of 19.3% in 2018 [4]. Since the availability and toxicity of the constituent elements in these technologies limit the production potential, the investigation of inexpensive and nontoxic absorbers with relevant optoelectronic properties becomes paramount [5,6,7,8,9,10,11,12].

In general, the theory suggests implementing two stable and phase-pure photosensitive materials exhibiting a bandgap (*E_g_*) of ~1.9 eV for the top and ~1.2 eV for the bottom photoelectrodes to approach the STH efficiency exceeding 23% for the D4 tandem cell [13]. Therefore, cheap and eco-friendly wide-bandgap metal oxides such as TiO_2_, BiVO_4_, or Fe_2_O_3_ are considered inappropriate for the bottom electrode, requiring a narrow bandgap semiconductor. Over the last decades, different cost-effective metal chalcogenides such as tin sulfide (SnS, *E_g_*~1.3 eV) [6,14] and copper-zinc-tin-sulfide (CZTS, *E_g_*~1.5 eV) [10,15] or metal oxide materials, e.g., CuFeO_2_ (*E_g_*~1.5 eV) [16] have been tried with limited success. Although the optoelectronic properties of CZTS give the impression of being suitable for the bottom photoelectrode, the low photocorrosion stability restrains its applicability in commercial PEC water splitting devices [10]. Thus, alternative semiconductors, which could meet the PEC water splitting process requirements, are pressingly needed to reach the practical STH efficiency [10,12].

Antimony (III) selenide (Sb_2_Se_3_) has recently emerged as an eco-compatible and low-cost absorber material for various photo-assisted applications due to its advantageous optoelectronic properties (*E_g_* ~1.2 eV, absorption coefficient > 10^5^ cm^−1^, high mobility) [7,17,18,19,20,21]. Besides, Sb_2_Se_3_ is internally stable towards photocorrosion, and its elemental composition suggests less-complicated chemistry within the device processing compared to the multielement compounds such as CIGS and CZTS [7,10,22]. All these signs indicate the great potential of Sb_2_Se3 for commercial high-performance PEC water splitting devices. However, in practice, Sb_2_Se_3_ tends to form a nanostructured morphology originating from its crystal structure rather than compact films [23], which requires unconventional strategies to improve the performance. Recently, compact thin-film Sb_2_Se_3_ photocathodes have demonstrated a record photocurrent density of 30 mA cm^−2^ at 0 V against the reversible hydrogen electrode (RHE) [22], even though the first value of 2 mA cm^−2^ at 0 V_RHE_ was reported in 2016 [23].

Despite significant efforts for improving the properties of typical absorber layers by various post-depositional treatments (PDTs) experienced for TFSCs [8,24,25,26,27,28], the procedures and their productivities of stable absorber/electrolyte interface are limited [29]. To date, most devices utilizing Sb_2_Se_3_ layers exhibited parameters significantly below expectations [17,18,19,20,21,22,30,31]. The current record photocurrent density for compact Sb_2_Se_3_ photoelectrodes has been predominantly attained by improvements in deposition methods in preference to realizing any suitable PDTs [22]. There have been some attempts to improve the structural properties of Sb_2_Se_3_ films by post-deposition selenization, yielding the solar cell efficiency of 6.06% that is still much below theoretical limits [31]. The moderate success achieved in the direction of Sb_2_Se_3_ designates that the synthesis, materials properties, device chemistry, and physics have yet been sufficiently understood. One way to improve materials properties is to implement the metal halide treatment, technologies with relatively comprehend mechanisms, and predicted results. The relative setbacks of alkali Me(I)Cl and alkaline earth Me(II)Cl metal chloride activation recently employed for Sb_2_Se_3_ materials and other absorber materials associated mostly with solid-state and vapor-phase transport processes which realized inhomogeneous doping of grains with some detrimental outcomes at the lattice scale [32,33]. Antimony (III) chloride as a logical choice for halide etching of Sb_2_Se_3_ with its strong tendency to hydrolysis and high volatility makes the activation procedure challenging to implement and almost not applicable. Thus, to involve metal halides and especially SbCl_3_ in the recrystallization and sintering of Sb_2_Se_3_, it is necessary to develop non-standard and subtle solutions that should ideally be simple and performed in air.

Here we report on a novel and simple approach that can be applied at ambient conditions and low temperatures to clean, recrystallize, sinter, and dope the grains of sputtered Sb_2_Se_3_ thin films. We implement a thermochemical SbCl_3_ treatment to fabricate smooth and phase-pure large-area (10 cm × 10 cm) Sb_2_Se_3_ photocathodes exhibiting a photocurrent density of around 31 mA cm^−2^ at −248 mV vs. SCE. During such treatment, Sb_2_Se_3_ thin films were dipped into glycerol solutions at various concentrations of SbCl_3_ and heated at 300 °C for different etching times, followed by 2 min of rinsing in deionized water. We also study the SbCl_3_-induced changes made to the structural and optoelectronic properties of Sb_2_Se_3_ films. We provide an intricate understanding of the multifunctional processes behind this thermochemical treatment. The postulated aspects can be valid not only for Sb_2_Se_3_ materials but also for other Sb-containing absorber materials.

## 2. Experimental Section

### 2.1. Synthesis of Sb_2_Se_3_ Layers

A commercially available antimony (III) selenide target (p.a. > 99.99%, Testbourne Ltd., London, UK) used for the fabrication of Sb_2_Se_3_ layers by Magnetron sputtering in an Evovac 030 inline sputter-coating system (Angstrom Engineering, Kitchener, ON, Canada). The target diameter was 4 inches. Sb_2_Se_3_ thin films were deposited onto Mo-coated and uncoated soda-lime glass substrates. During the deposition process, the substrate temperature was kept at 300 °C, the distance between the target and substrate was fixed as 20 cm, the pressure in the vacuum chamber was set to 5 × 10^−8^ Torr, the argon pressure was set to 5 mTorr, and plasma power was fixed as 88W.

### 2.2. SbCl_3_ Treatment

Antimony (III) chloride (p.a. > 99%, Sigma-Aldrich, Darmstadt, Germany), and glycerol (p.a. > 99.5%, Samaramedprod, Samara, Russia) were used as received. The Sb_2_Se_3_ films were dipped into 0, 50, 150, 300 and 450 mM solutions of SbCl_3_ in glycerol for etching times of 30, 60, and 90 min. The etching temperature was fixed at 300 °C, followed by 2 min of rinsing in deionized water.

### 2.3. Film Characterization

The structural properties of films were studied by X-ray Diffractometry (XRD, D8 Discover, Bruker, Leiderdorp, Netherlands, monochromatic Cu Kα_1_ radiation at 40 kV and 40 mA, a silicon strip detector D/teX Ultra) and Raman Spectroscopy (LabRam HR800 Horiba, Oberursel, Germany, the laser line at 532 nm, a spot diameter of 100 μm). The crystallite size of films was calculated using the Scherrer equation *D* = 0.94*λ*/*β*cos*θ*, where *λ* is the wavelength of the X-ray radiation (1.5406 Å), *θ* is the Bragg angle, and *β* is the full width at the half maximum (FWHM) of the peak located in the 2*θ* region of 16–18° in radians. The surface and cross-sections of films were investigated using scanning electron microscopy (SEM), and elemental compositions were studied by energy-dispersive X-ray (EDX) analysis (Merlin, Zeiss, Oerzen, Germany an acceleration voltage of 3 kV for SEM and 20 kV for EDX analysis). The elemental composition quantification was performed using PB/ZAF (Z-atomic number, A-absorption, and F-fluorescence) standard less mode. The film morphologies were probed using Atomic Force Microscopy (AFM, Bruker Nanoscope V controller, Leiderdorp, Netherlands with the application module MultiMode 8.10). The optical transmittance spectra of films were recorded in the wavelength range of 300–1500 nm at room temperature using a Cary 5000 UV-Vis-NIR spectrophotometer (Agilent Technologies, Inc., Santa Clara, CA, USA). The photoelectrochemical measurements were carried out using a Reference 3000 potentiostat/galvanostat (Gamry, Warminster, PA, USA) in a three-electrode cell configuration with a working electrode glass/Mo/Sb_2_Se_3_, a platinum wire counter electrode and a reference saturated calomel electrode (SCE, +0.244 V vs. SHE at 25 °C). The electrochemical experiments were performed using 0.1 M Na_2_SO_4_ solutions as a background electrolyte at pH of 6.3. The glass/Mo/Sb_2_Se_3_ electrodes were illuminated in electrochemical cells with light from a “white” LED lamp. The light intensity measured on the working electrode interface was around 30 mW cm^−2^.

### 2.4. Structure Fabrication and Characterization

For the preparation of Mo/treated-Sb_2_Se_3_/TiO_2_/IrO_x_ structures, Mo/Sb_2_Se_3_ layers were treated at 300 °C in 50 mM, 150 mM and 300 mM SbCl_3_-glycerol solutions for 90 min. Ultra-thin layers of TiO_2_ were deposited using a previously reported procedure [6], the 3Å-thick layers of IrO*_x_* were fabricated using magnetron sputtering deposition. The PEC performances of Mo/treated-Sb_2_Se_3_/TiO_2_/IrO_x_ electrodes were examined using 1 M H_2_SO_4_ solutions as a background electrolyte at pH below 1. The J–V curves were measured under a white light with an illumination intensity *P_SUN_* of 100 mW cm^–2^ (AM1.5). The half-cell solar-to-hydrogen (HC-STH) conversion efficiencies were calculated using the equation HC-STH = (I_PCD_*((*E_SCE_* + 0.248) − *E_H+_*_/H2_)/*P_SUN_*) * 100%, where *I_PCD_* is the photocurrent density obtained under an applied bias of *E_SHE_*, *E_H+/H2_* is 0 V_SHE_.

## 3. Result and Discussion

### 3.1. Morphological Studies

Post deposition thermal treatment is usually applied to absorber layers to improve the crystal quality and optoelectronic properties and increase device efficiency. Considerable grain growth and a decrease in the concentration of defects in bulk can be achieved at temperatures that promote loosening the crystal lattice (*T_L_*). This temperature point is roughly equal to half of the material’s absolute melting temperature, *T_L_*~305 °C for Sb_2_Se_3_. However, due to the high volatilities of Se and Se-comprising compounds possibly formed, treatments performed at temperatures exceeding T_L_ could generate conditions for the formation of selenium vacancies. In this study, Sb_2_Se_3_ layers were thermally treated in glycerol (GT) and SbCl_3_ glycerol-based media (Cl-GT) at temperatures slightly below *T_L_* to suppress the formation of Se vacancies and solvent decomposition.

**Figure 1:**
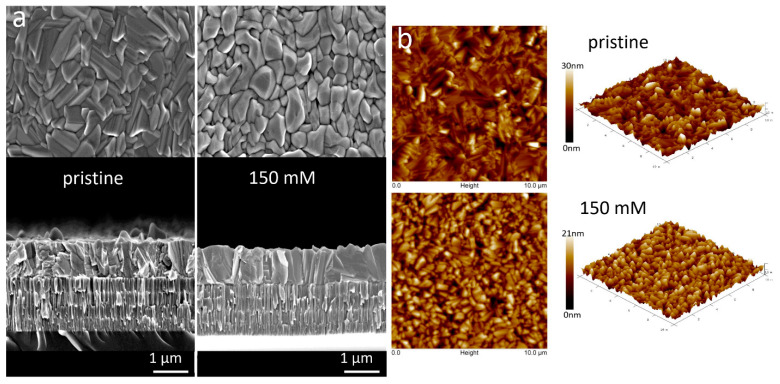
(**a**) SEM images, cross-sectional views and (**b**) AFM images of the pristine Sb_2_Se_3_ film and those thermally treated in glycerol at 150 mM SbCl_3_ for 90 min at 300 °C.

Figure 1 depicts SEM images, cross-sectional views and AFM images of the pristine Sb_2_Se_3_ film and those treated in glycerol at 150 mM SbCl_3_ for 90 min at 300 °C. The GT duration and SbCl_3_ concentration in glycerol affect the properties of Sb_2_Se_3_ films. The as-deposited films showed a dense structure with a wide grain-size distribution varying from 0.5 to 2 μm. The grains are randomly orientated to the substrate and have plate-like shapes with sharp edges (Figure 1a). Preliminary experiments enclosed that GT of films for 90 min is more effective for material recrystallization than less-time-consuming process. The Sb_2_Se_3_ films began to form a pillar-like structure and were still attached to the substrate after GT for 90 min (Supplementary Figure S1), while longer processing time in glycerol yielded the formation of cracks and then to the complete destruction of Sb_2_Se_3_ layers after GT for 120 min. The thermal treatment for 90 min in glycerol containing SbCl_3_ modified the morphology of Sb_2_Se_3_ films considerably. The Cl-GT at 50 mM yields to the film consisted of faceted-shaped grains with rounded edges. The film has a nearly columnar structure with some smaller grains disposed additionally near to the Mo substrate (Supplementary Figure S2). In the presence of 150 mM SbCl_3_, Sb_2_Se_3_ films experience unusual grain sintering to develop grains with a size larger than the film thickness and with significantly decreased the concentration of grain boundaries (GBs) (Figure 1a). It should be noted that a further increase in the concentration of SbCl_3_ to 300 and 450 mM provided a reverse evolution for morphologies of Sb_2_Se_3_ films, resulting in morphological properties like that observed for films after 50 mM Cl-GT (Supplementary Figure S2). It is evident that Cl-GT at 150 mM significantly enhances the grain size of Sb_2_Se_3_ films, decreased the density of GBs, resulting in films with a close-packed morphology compared to other concentrations tried.

To confirm the observed surface changes, we also examined the morphologies of Sb_2_Se_3_ films by AFM (Figure 1, Supplementary Figure S3). Phase and topographic AFM analysis verified the morphological characteristics revealed from SEM. The AFM images show that the pristine film is granular, suggesting island growth. Moreover, there is a bimodal height distribution amongst the plate-like grains, where the taller grains are dispersed and disorderly distributed within the film. From the AFM phase and 3D images in Figure 1b, it is seen that the shape of grains changed to a rounded and smooth one, and the grain distribution become more homogenous over the surface after the Cl-GT (Supplementary Figure S3). A drastic discrepancy between as-deposited Sb_2_Se_3_ films and those thermally treated at various concentrations of SbCl_3_ in glycerol is the film roughness. The roughness increased slightly from 30 nm for as-deposited films to 33 nm for 50 mM Cl-GT films. Then it was considerably decreased to 21 nm for the films treated at 150 mM SbCl_3_, mostly due to denser and larger grains obtained at this concentration. Finally, roughness measured for 300 mM and 450 mM Cl-GT films were 30 nm and 38 nm, respectively (Table 1). The reverse effect observed at higher concentrations of SbCl_3_ in glycerol is probably related to the liquid media’s densification and its deactivation.

### 3.2. Compositional Analysis

The EDX studies revealed a slight Se-deficiency in the as-deposited film compared to stoichiometric Sb_2_Se_3_ materials (Table 1). No apparent changes in the thickness and elemental composition of Sb_2_Se_3_ films after GT at any duration, and no carbon contaminations are detected (Supplementary Figure S4). It was surprising that Cl-GT introduces some deviation in elemental compositions of films. According to the EDX analysis, a slight Se-deficiency observed in as-deposited films disappeared after the 150 mM Cl-GT, yielding the formation of Sb_2_Se_3_ films with a stoichiometric composition. It seems that Cl-GT at specific concentrations assists in dissolving the excess of Sb from Sb_2_Se_3_ films and by this enables the formation of stoichiometric film composition through the several steps:

SbCl_3_ + HOCH_2_–CH(OH)–CH_2_OH ⇄ Sb(C_3_O_3_H_4_) + 3HCl (1)

HOCH_2_–CH(OH)–CH_2_OH + HCl → HOCH_2_–CH(OH)–CH_2_Cl + H_2_O (2)

HOCH_2_–CH(OH)–CH_2_Cl → H_2_C=CH–CHO + HClO + H_2_O (3)

HClO + HCl → Cl_2_ + H_2_O (4)

Sb_(2+*x*)_Se_3_ + 3*x*/2 Cl_2_ → *x*SbCl_3_ + Sb_2_Se_3_ (5)

Some minor deviation in stoichiometry was also observed for Sb_2_Se_3_ films thermally treated at 300 mM SbCl_3_ in glycerol (Table 1). The lack of Se in the films turned back after the 450 mM Cl-GT. The observed changes in morphological and compositional properties of films can be explained by the densification of solutions taking place at higher concentrations of SbCl_3_ and discussed later.

### 3.3. Structural Analysis

**Figure 2:**
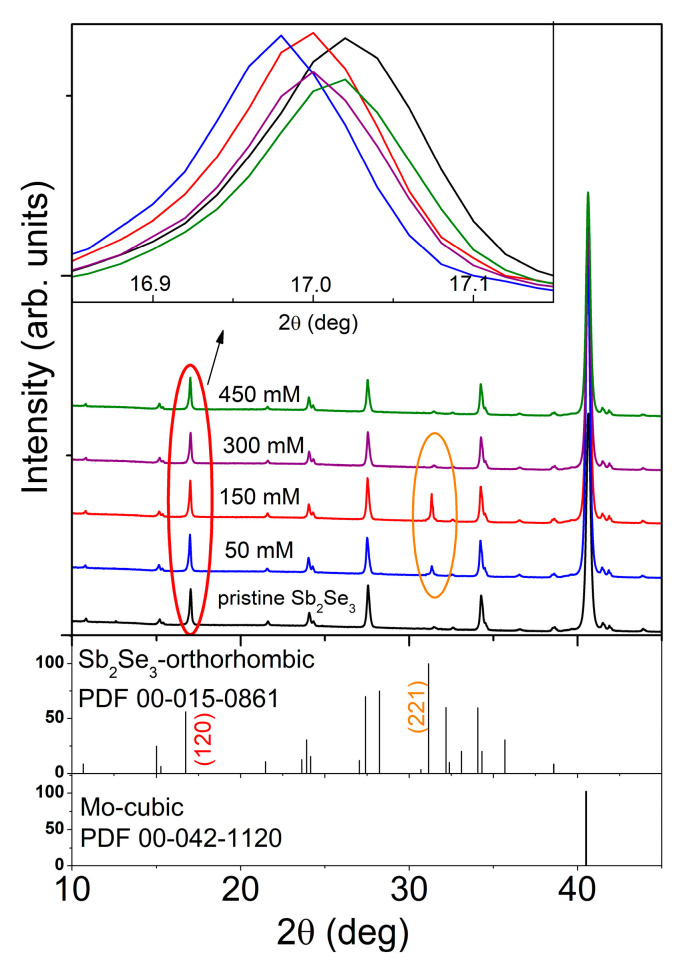
XRD patterns of the pristine Sb_2_Se_3_ film and those thermally treated in glycerol at various concentrations of SbCl_3_ for 90 min at 300 °C.

Figure 2 compares the XRD patterns of Sb_2_Se_3_ films sputtered on Mo-coated glass substrates before and after Cl-GT procedures. For each sample, the most intense diffraction peak belonged to the Mo substrate. All the diffractograms showed a crystalline orthorhombic Sb_2_Se_3_ phase (PDF card no: 00-015-0861) represented mainly by four prominent (120), (101), (230) and (420) Bragg reflections. The XRD patterns demonstrated no presence of elemental Sb, Se, or any oxide secondary phases and no obvious alterations for Sb_2_Se_3_ films after Cl-GT. In all cases, no clear crystallographic preferred orientations were detected (Supplementary Table S1). We also noted that Sb_2_Se_3_ starts to recrystallize along the (221) Bragg reflection when 50 mM SbCl_3_ solution was used. The diffraction peak intensity intensified with increasing the concentration of SbCl_3_ to 150 mM and then diminished after the processing at 300 mM and 450 mM SbCl_3_. Thus, the 150 mM Cl-GT could yield compact Sb_2_Se_3_ thin film with vertically oriented (Sb_4_Se_6_) ribbons in the (221) plane that could potentially make the separation of charges more efficient [22].

In order to learn about the potential influence of the novel thermochemical treatment, a detailed structural analysis was performed. We observed that the crystallite size increased from 73 nm for the pristine film to 83 nm and then to 95 nm for 50 mM and 150 mM Cl-GT Sb_2_Se_3_ films, respectively (Table 1). Further increasing the concentration of SbCl_3_ in glycerol to 450 mM decreased the crystallite size to 75 nm, to the value that was similar to the pristine film value. In the meantime, we noticed that the density of the glycerol system increased at higher concentrations of SbCl_3_; the visual inspection confirmed the change in the system from transparent and homogenous to red-colored and dense solutions. This fact indicated that SbCl_3_ concentrations exceeding 150 mM represented the critical conditions in which the reactivity of the system significantly decreased. As SbCl_3_ acts as a Lewis acid, a denser system can be formed upon the evolution of acrolein (H_2_C=CH–CHO, Equation (3)) and its polymerization. This, in turn, might decrease the activity of SbCl_3_.

The interesting behavior was recorded when we carefully analyzed the XRD data of obtained films. The lattice parameters of Sb_2_Se_3_ films treated in 50 mM SbCl_3_ solution increased significantly along with all three a, b, and c axes. Thermochemical treatment at higher concentrations of SbCl_3_ in glycerol yielded a systematical decrease in lattice parameters (Table 1). This phenomenon partnered with a shift of, e.g., the (120) reflection (Figure 2) and deviations in elemental compositions (Table 1, Supplementary Figure S4). After Cl-GT at 50 mM SbCl_3_, a close inspection revealed an offset of the (120) XRD peak toward lower 2*θ* values, conforming to an increase in the lattice constants.

Considering the results of EDX studies, the observed effect was expected and associated mainly with the formation of more stoichiometric film composition (Table 1). Upon the treatment in 150 mM SbCl_3_ solution, the film composition remained still stoichiometric, while the (120) peak shifted back to higher 2*θ* values, indicating a decrease in lattice parameters. As the ionic radius of Cl^−^ (1.81 Å) is smaller than that of Se^2−^ (1.98 Å) [25] and chlorine has a higher electronegativity than selenium, the observed contraction of the Sb_2_Se_3_ lattice can be probably attributed to the incorporation of chlorine at selenium sites accompanied by the formation of antimony vacancies. Such incorporation/substitution phenomena are relatively common and have been previously observed in SnCl_2_-annealed SnS films and CdTe films after the required activation in CdCl_2_ [8,26]. The changes in the lattice constants of Sb_2_Se_3_ films introduced by the treatment at 300mM and 450 mM SbCl_3_ are mostly associated with the returned selenium deficiency (Table 1).

**Figure 3:**
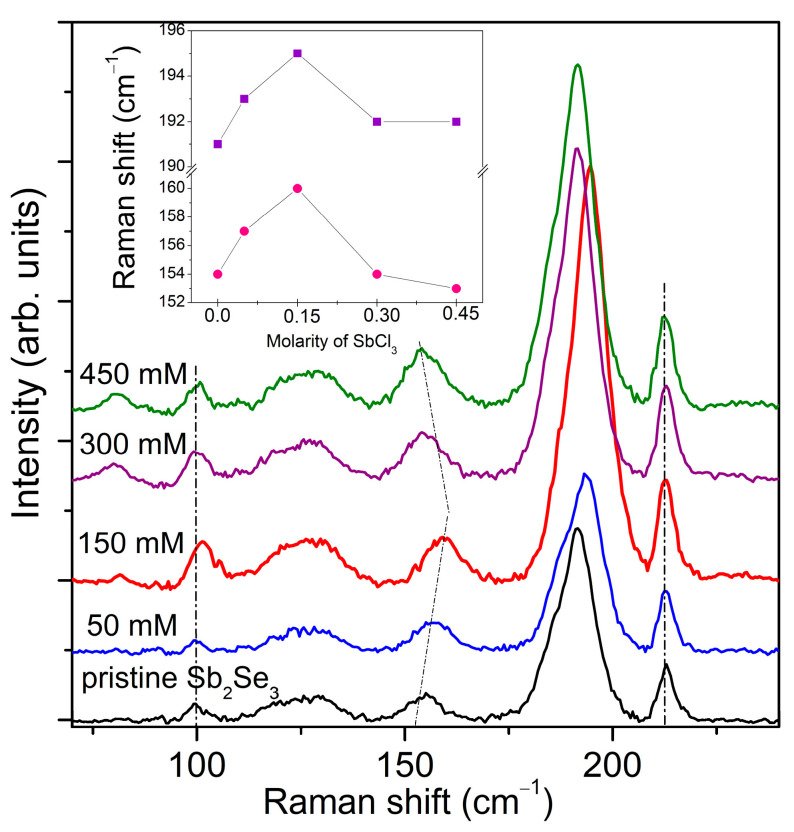
Raman spectra of the pristine Sb_2_Se_3_ film and those thermally treated in glycerol at various concentrations of SbCl_3_ for 90 min at 300 °C.

We applied Raman spectroscopy to probe the effect of metal halide thermochemical treatment and investigate further the compositional properties of films. The corresponding Raman spectra of the pristine and treated films are shown in Figure 3. The Raman spectrum of the pristine Sb_2_Se_3_ film displays four prominent peaks at 99, 155, 191, and 213 cm^−1^ commonly assigned to the orthorhombic Sb_2_Se_3_ phase [34] and the weak intensity band at 252 cm^−1^ attributed to the presence of trace amounts of antimony oxide (Supplementary Figure S4) [35,36]. A distinguishable shift in the relative peak positions towards higher energies was detected for the films thermochemically treated in 50 and 150 mM SbCl_3_ solutions (Figure 3). The observed blue shift of the peaks with original positions at 155 and 191 cm^−1^ can be related to the reduced level of internal stress in the films as Sb excess was disappeared and crystalline quality improved after the Cl-GT. Similar changes in peak positions have been recently reported for SnS films when the Sn/S ratio was increased and moved towards the more stoichiometric values [37]. With increased concentrations of SbCl_3_ in solution to 300 mM, a reverse red shift was observed, indicating the back evolution of tensile strain (Figure 3, Table 1). Such unusual behavior of the characteristic Raman modes at 155 and 191 cm^−1^ ought to correlate with the specific atoms situated at the site of these vibrational modes. Isotropic changes in the lattice parameters of orthorhombic Sb_2_Se_3_ caused by fluctuations in the film composition determine alterations in the bond length, reflecting in some variations of stretching force constants. This generates an indirect correlation with lattice constants since the phonon energy is proportional to the force constants. It needs to be noted that the pristine film and those processed in 450 mM SbCl_3_ had almost identical lattice parameters (Table 1). Considering this fact, it was assumed that the bond length and force constants should be very similar for both cases. Thus, the films should have exhibited a slight difference in Raman peak positions in theory, and it was observed in practice (Figure 3). It was also surprising that the weak vibrations belonging to antimony oxide disappeared after the treatments performed in 50, 150, and 300 mM SbCl_3_ solutions and appeared again when 450 mM solution was used for processing (Supplementary Figure S5).

### 3.4. Optical Data Analysis

We further explored the optical behavior of the pristine and treated Sb_2_Se_3_ films using the optical bandgap values estimated from optical transmittance data using the well-known Tauc equation [25] and grouped in Table 1. Consequently, we discovered that the bandgap values of obtained films were particularly sensitive to the concentration of SbCl_3_ in glycerol. Although the films exhibited no sharp linear Tauc plots, the trend corresponded to changes in the lattice parameters caused by elemental composition deviations. Thus, obtained bandgap values were trustworthy (Supplementary Figure S6). According to the optical data analysis, the pristine films exhibited two bandgap values such as 1.30 eV corresponding to the orthorhombic Sb_2_Se_3_ phase [38] and 1,21 eV that might be related to the Sb-rich oxyselenide phase (Sb_2_O_5_ *Eg* = 0.76 eV) [35,38]. This was in good agreement with the EDX and Raman data indicating the presence of some antimony oxide phase (Supplementary Figures S4 and S5). The bandgap of Sb_2_Se_3_ films treated in the 50 mM SbCl_3_ solution decreased slightly to 1.27 eV as some Sb excess was detected. Treatment performed in the 150 mM SbCl_3_ solution yielded the stoichiometric films with the *Eg* value of 1.34 eV. In general, Sb_2_Se_3_ films with stoichiometric compositions have demonstrated higher bandgap values than those with some selenium deficiency [38]. The thermochemical processing at 350 and 450 mM SbCl_3_ lowered the direct bandgap values to 1.32 eV, accompanied by reverse increasing Sb content [38]. It should also be noted that a second bandgap at 1.22 eV corresponding to the earlier mentioned Sb-rich oxyselenide phase repeatedly appeared in the 450 mM SbCl_3_-treated film (Table 1, Supplementary Figure S6).

### 3.5. Photoelectrochemical (PEC) Analysis

**Figure 4:**
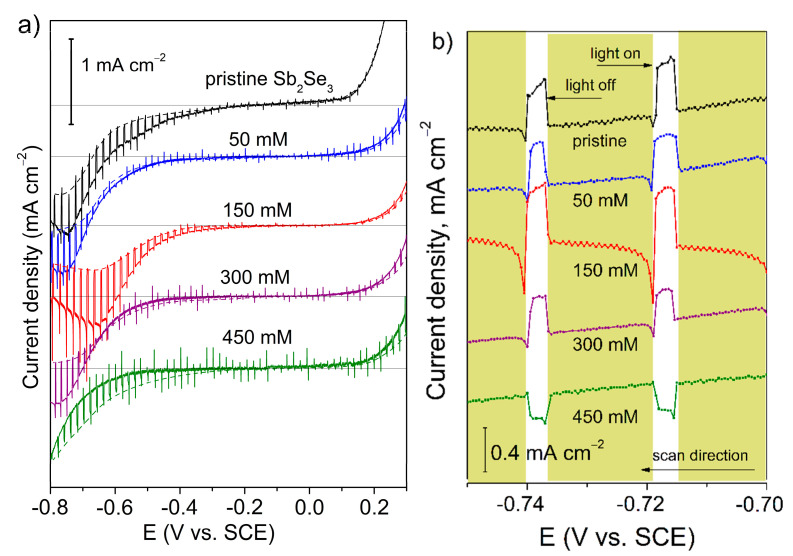
(**a**) Electrochemical characteristics of the pristine and thermally treated Sb_2_Se_3_ films measured in the dark (dash line) and under chopped illumination (solid line) in the 0.1 M Na_2_SO_4_ supporting electrolyte. (**b**) The enlarged photoelectrochemical characteristics recorded in the potential region from −0.70 V to −0.75V vs. SCE.

For further development of successive technologies to fabricate complete high-performance water splitting cells, we characterized additionally the surface properties of pristine and SbCl_3_-etched Sb_2_Se_3_ films using photoelectrochemical (PEC) analysis as it was suggested in [10]. All the formed films were estimated using linear-sweeping current–potential (*I–V*) scans at a sweep rate potential of 20 mV s^−1^ in a 0.1 M Na_2_SO_4_ electrolyte under a chopped illumination from a white LED lamp. Figure 4a shows the PEC behavior across the potential range from 0.3 V to −0.8 V vs. SCE in the negative directions. Pristine and treated films exhibited the forward current at positive potentials and very low reverse current at negative potentials, corresponding to a Schottky barrier between p-type semiconductor and electrolyte [6,10]. The open-circuit voltage (*V_OC_*) of the pristine film was found to be −0.13 V vs. SCE (0.49 V vs. RHE, Supplementary Equations (S1) and (S2)), while the *V_OC_* values of treated Sb_2_Se_3_ films shifted toward more positive potentials of about −0.05 V vs. SCE (0.57 V vs. RHE, Supplementary Equation (S3)). For most Sb_2_Se_3_-based photoelectrodes, *V_OC_* values less than 0.5 V vs. RHE have been typically reported [20,21,22,23]. This *V_OC_* deficiency could be related to different material aspects such as (a) low quality of the single-phase Sb_2_Se_3_, a high concentration of defects and grain boundaries along with the presence of secondary crystalline or amorphous phases, which are harmful to the device performance [8,20,21,22,23]; (b) insufficient structure optimization, e.g., lattice mismatch, and an inauspicious band offset between p-type Sb_2_Se_3_ and n-type CdS grown using a chemical bath [22]. Several authors reported the *V_OC_* values of about 0.6 V vs. RHE measured in Na_2_SO_4_ electrolytes with pH values lying between 5 and 6.5 [39,40]. Thus, the *V_OC_* values observed for the pristine film and those that experienced the thermochemical treatment developed in this work agreed with the flat-band potential of about 0.5–0.7 V vs. RHE [39,40,41] and *V_OC_* data reported for p-type Sb_2_Se_3_ photocathodes [39,40]. Irrespective of the etching conditions employed, the photocurrent increased with increasing cathodic polarization due to increased band bending. The existence of a dark current below *Voc* demonstrated the presence of some impurities on the semiconducting surface. The pristine Sb_2_Se_3_ film revealed a higher dark current than that recorded for etched films. In theory, a defect-free surface of a p-type semiconductor shows no dark reduction current unless high overpotentials are applied. Thus, we concluded that the pristine film and 450 mM treated film possessed more defective surfaces. In contrast, etching in 50, 150 and 300 mM SbCl_3_ glycerol solutions decreased various imperfections such as Sb-rich oxyselenide phase, GBs, and some other possible defects presented in films. This was supported by the EDX (Table 1, Supplementary Figure S4) and Raman (Supplementary Figure S5) data on the presence of oxide phase and some selenium-deficiency found in the pristine and 450 mM SbCl_3_ etched Sb_2_Se_3_ films, and SEM and AFM studies displaying more compact morphology for the film after 150 mM Cl-GT (Figure 1b, Supplementary Figures S1–S3). The dark current of all samples increased at potentials below −0.45 V, indicating the reduction of semiconducting surfaces via several electrochemical reactions:

Sb_2_O_5_ + 4H^+^ + 4e^−^ ⇄ Sb_2_O_3_ + 2H_2_O (6)

Sb_2_O_3_ + 6H^+^ + 6e^−^ ⇄ 2Sb + 3H_2_O (7)

Sb_2_Se_3_ + 3H^+^ + 6e^−^ ⇄ 2Sb + 3HSe^−^   −0.65 V vs. SCE (8)

Under illumination, the photocurrent of all Sb_2_Se_3_ films intensified with increasing cathodic polarization reaching the maximum at −0.70 V. Figure 4b enlarges the photoelectrochemical characteristics in the region from −0.70 V to −0.75 V vs. SCE for better resolution of photocurrent transient. The 150 mM Cl-GT increased the film sensitivity and obtained maximum photocurrent, indicating the highest ability for photon-to-electron conversion. The photocurrent maximum of 150 mM SbCl_3_-etched film shifted to more negative potentials, suggesting the formation of homogeneous and phase-pure p-type material that contains minimum impurities accumulating the charge (Figure 4a). In general, the conductivity type of materials can be tuned by intrinsic and extrinsic point defects. It is known that cation vacancies exhibit low stability despite the formation of acceptor type states, and the chlorine tended to occupy interstitial sites and behave as amphoteric impurity [32]. At concentrations of SbCl_3_ in glycerol between 50 and 150 mM, chlorine serving as a shallow donor seemed to occupy the selenium site of Sb_2_Se_3_, analogously to the models of Cl_Te_ and Cl_S_ accepted for CdTe and SnS [8,26]:

Sb_2_Se_3_ + *n*SbCl_3_ ⇄ [(1+*n*)Sb_Sb_Se_Se_*n*V_Sb_(3*n*)Cl_Se_] (9)

Our theoretical calculations revealed low formation energies for the defect defect-impurity complex V_Sb_-Cl_Se_ having the potential to provide p-type conductivity for Sb_2_Se_3_ (Supplementary Table S2). It was surprising that a further increase in the concentration of SbCl_3_ to 300 mM led to the photocurrent inversion under illumination; the current became less than the dark current in the potential region from 0 V to −0.6 V vs. SCE (Figure 4a). This effect was more noticeable for the film treated at 450 mM SbCl_3_ and could be associated with the formation of an n-type dense layer of Sb-rich oxyselenide on the film surface (Figure 4b, Table 1, Supplementary Figures S3–S5). Considering the EDX, UV-Vis, Raman, and PEC data, it seems that detrimental Sb-rich phase and antimony oxide could be effectively eliminated from Sb_2_Se_3_ films by the simple etching at 150 mM SbCl_3_ through (Equation (5)) and the reaction:

Sb_2_O_3_ + 6HCl → 2SbCl_3_ + 3H_2_O (10)

It should also be noted that p-type conductivity can be enhanced and stabilized by the novel thermochemical treatment proposed for polycrystalline Sb_2_Se_3_ films.

### 3.6. PEC Performance of Treated Sb_2_Se_3_ Photocathodes

**Figure 5:**
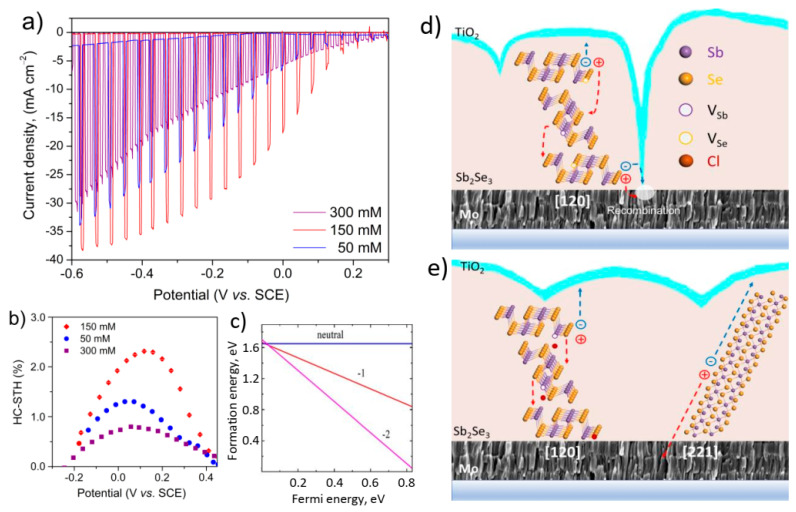
(**a**) *J-V* curves for Mo/TR-Sb_2_Se3/TiO_2_/IrOx electrodes recorded in 1M H_2_SO_4_ electrolytes using a sweep rate potential of 20 mV s^–1^ from positive to negative potential. (**b**) HC-STH efficiencies. (**c**) Formation energy for the V_Sb_-Cl_Se_ complex forming acceptor states within the bandgap. (**d**,**e**) Recombination of charge carries, possible ways for passivation of GBs and charge separation along the planes.

To estimate the applicability of the novel metal halide thermochemical treatment to improve photoelectrochemical material properties, we completed the photocathode structures at the configuration of Mo/treated-Sb_2_Se_3_/TiO_2_/IrO_x_ (Figure S7 Supplementary demonstrates a schematic route for fabrication). Figure 5a demonstrates the PEC performances of 50 mM, 150 mM and 300 mM SbCl_3_-derived Sb_2_Se_3_ photocathodes measured in acidic solutions with pH values around 1. The on-set potential of photocurrent shifted towards a positive direction after increasing the concentration of SbCl_3_ from 50 to 150 mM, and then shifted back towards a negative direction when 300 mM SbCl_3_ treatment was employed. This behavior can be associated with a less-defective surface formed upon the 150 mM activation [6,10]. The 50 mM Cl-GT electrode revealed the PCD values of around 21 mA cm^−2^ at −248 mV vs. SCE, while PCD of the 150 mM-activated sample approximated to ~31 mA cm^−2^ at −248 mV vs. SCE, the highest value yet observed for Sb_2_Se_3_ photoelectrodes [12,22]. The simplicity of both synthesis and novel thermochemical treatment yielding to high PCD clearly illustrates a big potential of the proposed protocol to fabricate efficient Sb_2_Se_3_ photocathodes.

The HC-STH conversion efficiencies derived from the third scans of the 50 mM, 150 mM and 300 mM Cl-GT electrodes are compared in Figure 5b. It is worth noting that the 150 mM sample exhibited HC-STH values higher than those observed for 50 mM and 300 mM Cl-GT samples. Furthermore, the maximum HC-STH value of 150 mM treatment appeared at a more positive potential of 135 mV vs. SCE in contrast to 50 mM and 300 mM analogs (50 mV vs. SCE). This fact indicates that activating Sb_2_Se_3_ layers in the presence of SbCl_3_ at concentrations of around 150 mM is a beneficial strategy to improve the photocurrent, photovoltage and fill factor. The difference in electrode performances originated from two main aspects: (a) the 50 mM and 300 mM-treated samples demonstrated rough topography (Supplementary Figure S3) along with poor stoichiometry (Table 1) and probably higher and less-homogeneous surface potential at GBs [22,42], while the 150 mM-activated electrode offered stoichiometric composition, uniform morphology (Figure 1b), and therefore lower surface potential at GBs [22,42]; (b) the novel thermochemical protocol seemed to be efficient to passivate the GBs alone (hk0) planes, e.g., the (120) plane via the formation of the defect-impurity V_Sb_-Cl_Se_ complex that could form two shallow acceptor states in the bandgap with the low energies of 18 meV for (0/−1) and 37 meV for (−1/−2) (Figure 5c). Furthermore, the 150 mM Cl-GT could initiate the recrystallization of Sb_2_Se_3_ along the (221) plane (Figure 2). Yang et al. reported that surface potential raised considerably with an increase in layer roughness (Table 1), revealing direct contact between n-type TiO_2_ and metallic substrate due to pinholes [22]. Under this scenario, the back Mo contact could act as a recombination center for photogenerated electrons owing to the electric field at the p-n junction (Figure 5d). Possible direct contact between TiO_2_ and Mo, accompanied by some deviations in phase compositions and optoelectronic properties, led to worse performance of the 300 mM-treated electrode than that derived from the 50 mM SbCl_3_ solution.

By contrast, the more compact 150 mM-treated sample with phase-pure composition and optimal optoelectronic properties revealed the highest performance. The photoexcited charge carriers in this electrode could separate more efficiently at the passivated (120) plane and along the [Sb_4_Se_6_] ribbons of the (221) plane (Figure 5e) due to the p-n junction and decreased surface potential.

## 4. Conclusions

Experimental confirmations were reported to support efficient removal of antimony-rich oxyselenide phase(s) presented in sputtered Sb_2_Se_3_ absorbers using a novel thermochemical treatment in glycerol solutions containing SbCl_3_. The SEM-EDX, Raman, UV-Vis and PEC analyses performed on treated films revealed the grain growth, sintering, and the removal of secondary phases from the bulk when 150 mM SbCl_3_ solution was used. Furthermore, PEC measurements have demonstrated that the exposure of Sb_2_Se_3_ into Cl-GT solutions at 300 °C for 90 min yields enhancing photoelectrochemical performance and the HC-STH efficiency up to 2.4%. It is believed that chlorine tends to incorporate into Sb_2_Se_3_ at the selenium site, promoting the formation of p-type conductivity. The proposed simple thermochemical process opens a novel route for the selective removal of secondary phases and, by this, can contribute to improvements in the performance of antimony-based absorber solar cells. The low toxicity of reagents used, and the simplicity of the procedure give additional interest to implementing this route in other PV technologies.

## Figures and Tables

**Table 1:** The atomic percentages of antimony and selenium according to the EDX data, the arithmetic average of the absolute values of the surface height deviations (*R_a_*) according to the AFM data, optical direct band gap values (*E_g_*), the average crystallite size (D), and lattice parameters of Sb_2_Se_3_ films depending on the molarity (M) of SbCl_3_ in glycerol. Each parameter was determined by averaging the values obtained three measurements for each sample. The error represents the standard deviation.

Treatment	*M* (mM)	Elements (at.%)		*Ra*	*E_g_* (eV)	*D* (nm) (±0.7)	Lattice Parameters (Å) (±0.001)		
		Sb	Se	(nm)			*a*	*b*	*c*
**pristine**		42.1	57.6	30	1.30 (1.21)	73	11.591	11.729	3.901
SbCl_3_-glycerol	50	40.8	59.2	33	1.27	83	11.612	11.760	3.973
	150	40.1	59.9	21	1.34	95	11.594	11.733	3.907
	300	40.4	59.6	30	1.32	83	11.592	11.731	3.903
	450	42.0	57.9	38	1.32 (1.22)	75	11.591	11.730	3.902

## Data Availability

The data presented in this study are openly available in Mendeley Data, V1, doi: 10.17632/gwpzmr2fs7.1.

## Supplementary Materials

The following are available online at https://www.mdpi.com/2079-4991/11/1/52/s1, Figure S1: SEM images of the pristine Sb_2_Se_3_ film and those thermally treated in glycerol at 300 °C for 30 and 90 min, Figure S2: SEM images of Sb_2_Se_3_ films thermally treated in glycerol at 50, 300 and 450 mM SbCl_3_ for 90 min at 300 °C, Figure S3: AFM images of Sb_2_Se_3_ films thermally treated in glycerol at 50, 300 and 450 mM SbCl_3_ for 90 min at 300 °C, Figure S4: EDX spectra of the pristine Sb_2_Se_3_ film and those thermally treated in glycerol at various concentrations of SbCl_3_ for 90 min at 300 °C, Figure S5: Raman spectra of the pristine Sb_2_Se_3_ film and those thermally treated in glycerol at 450 mM SbCl_3_ for 90 min at 300 °C, Figure S6: Tauc plots of the pristine Sb_2_Se_3_ film and those thermally treated in glycerol at various concentrations of SbCl_3_ for 90 min at 300 °C, Figure S7: A sketch for the fabrication of Mo/treated-Sb_2_Se_3_/TiO_2_/IrO_x_ structures along with an electrochemical cell under solar illumination, Table S1: Ratios of intensities of (iii) and (221) diffraction peaks for the pristine and treated Sb_2_Se_3_ samples, Table S2: Neutral defects formation energies.
