# Supplementary material for: A Novel Thermochemical Metal Halide Treatment for High-Performance Sb2Se3 Photocathodes
Source: Nanomaterials (Basel). 2020 Dec 28;11(1):52. doi: 10.3390/nano11010052 (PMC7824267; doi:10.3390/nano11010052)
Supplement: Supplementary file 1 [file nanomaterials-11-00052-s001.pdf]

nanomaterials

Supplementary Materials

A Novel Thermochemical Metal Halide Treatment for High-Performance Sb_2Se_3 Photocathodes

Svetlana Polivtseva [1,*], Joseph Olanrewaju Adegite [2], Julia Kois [3], Damir Mamedov [4,5], Smagul Zh. Karazhanov [4,5], Jelena Maricheva [1] and Olga Volobujeva [1]

[1] School of Engineering, Department of Materials and Environmental Technology, TalTech, Ehitajate tee 5, 19086 Tallinn, Estonia; jelena.maricheva@taltech.ee (J.M.); olga.volobujeva@taltech.ee (O.V.)
[2] Mechanical Engineering Department, Worcester Polytechnic Institute, 100 Institute Road, Worcester, MA 01609, USA; joadegite@wpi.edu
[3] LLC Auramet, Kalliomäentie 1B, 02920 Espoo, Finland; juliakois2@gmail.com
[4] Department of Materials Science, National Research Nuclear University (MEPhI), 115409 Moscow, Russia; damir.mamedov@ife.no (D.M.); smagulk@ife.no (S.Z.K.) damir.mamedov@ife.no (D.M.); smagulk@ife.no (S.Z.K.)
[5] Department for Solar Energy, Institute for Energy Technology, NO-2027 Kjeller, Norway
* Correspondence: cvpolcv@gmail.com

Citation: Polivtseva, S.; Olanrewaju, J.A..; Kois, J.; Mamedov, D.; Karazhanov, S.Z.; Maricheva, J.; Volobujeva, O. Novel Thermochemical Metal Halide Treatment to High-Performance Sb_2Se_3 Photocathode. *Nanomaterials* **2021**, *11*, 52. https://doi.org/10.3390/nano11010052

Received: 22 November 2020
Accepted: 24 December 2020
Published: 28 December 2020

Publisher's Note: MDPI stays neutral with regard to jurisdictional claims in published maps and institutional affiliations.

Copyright: © 2020 by the authors. Licensee MDPI, Basel, Switzerland. This article is an open access article distributed under the terms and conditions of the Creative Commons Attribution (CC BY) license (http://creativecommons.org/licenses/by/4.0/).

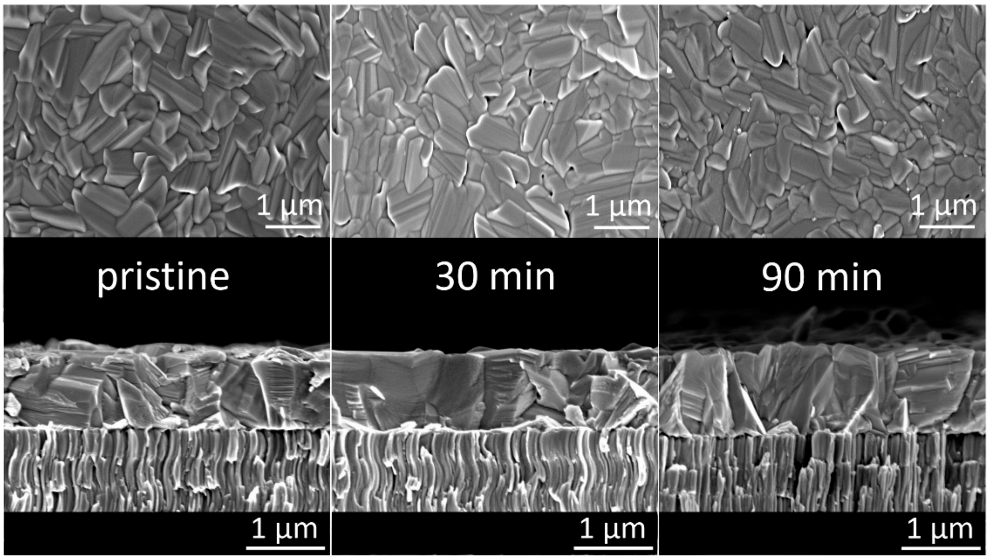

Figure S1. SEM images of the pristine Sb_2Se_3 film and those thermally treated in glycerol at 300 °C for 30 and 90 min.

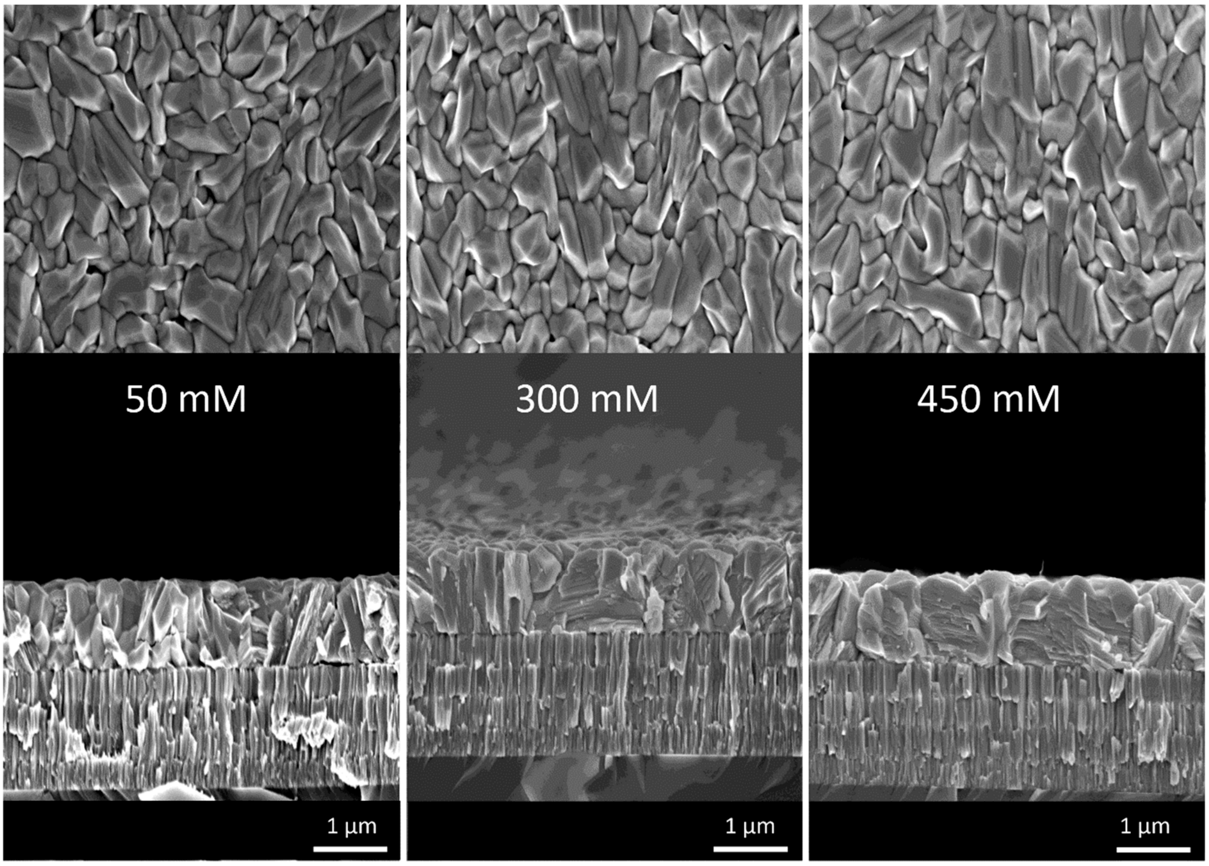

Figure S2. SEM images of Sb_2Se_3 films thermally treated in glycerol at 50, 300 and 450 mM $SbCl_3$ for 90 min at 300 °C.

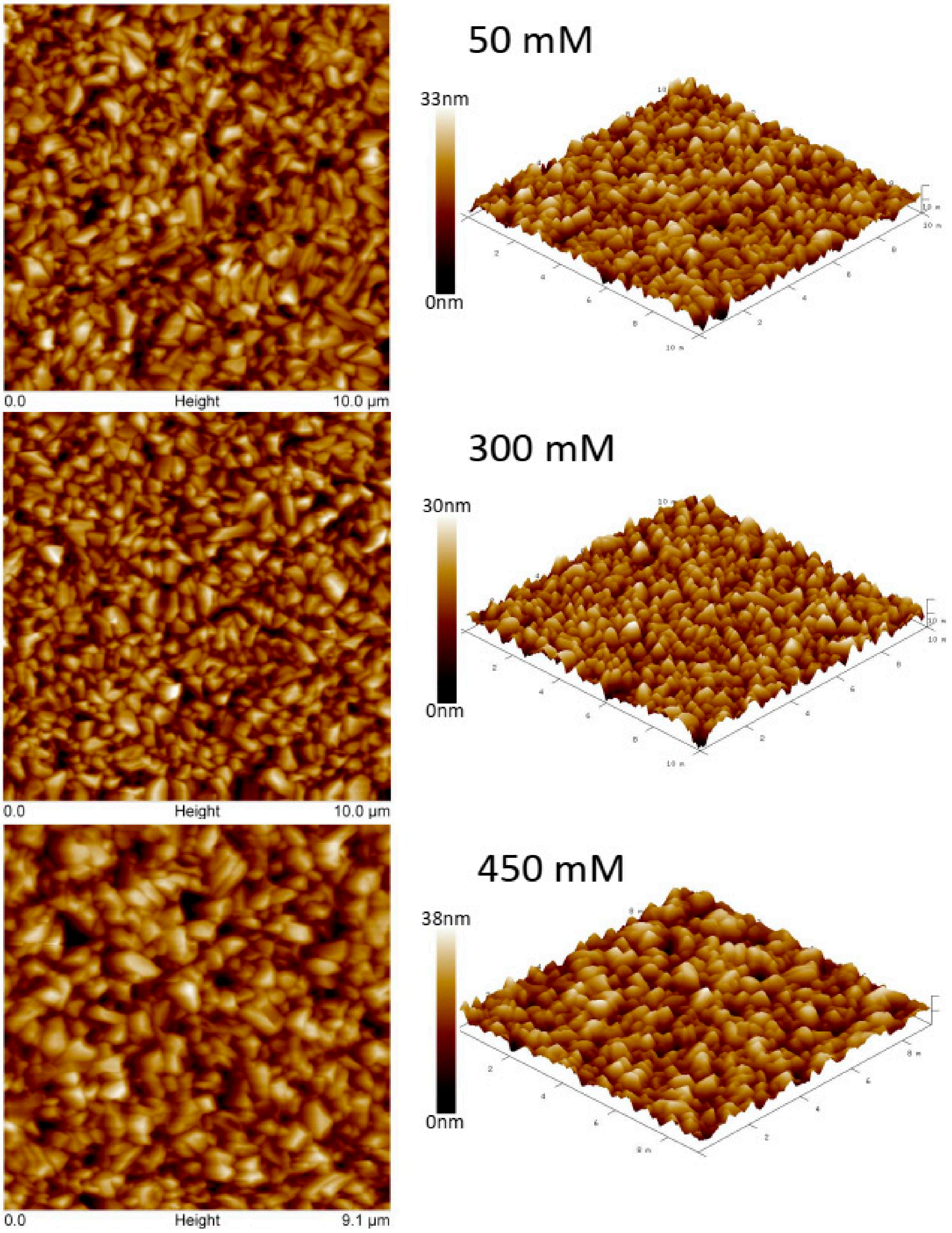

Figure S3. AFM images of Sb_2Se_3 films thermally treated in glycerol at 50, 300 and 450 mM $SbCl_3$ for 90 min at 300 °C.

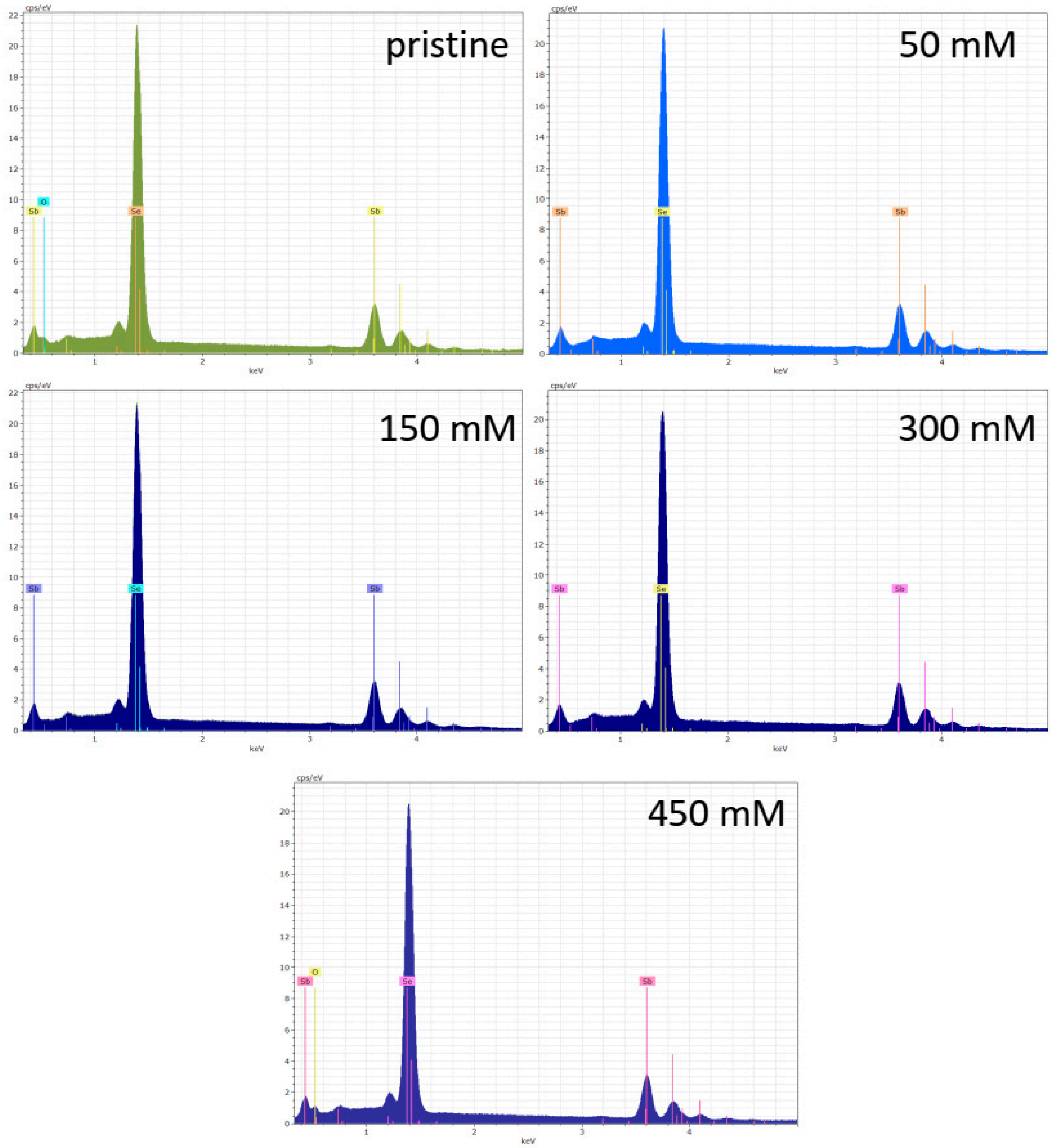

Figure S4. EDX spectra of the pristine Sb_2Se_3 film and those thermally treated in glycerol at various concentrations of $SbCl_3$ for 90 min at 300 °C.

Orientation of crystallites

Detailed comparison of the recorded diffractograms with the reference file (PDF 01-083-1758) for orthorhombic Sb_2Se_3 powder revealed no preferred orientation of crystallites in the pristine and treated Sb_2Se_3 films. The (221) XRD reflection at the 2θ value of 31.16° is the highest reflection for the Sb_2Se_3 powder. The pristine Sb_2Se_3 film and those treated in $SbCl_3$-glycerol media exhibited four main (120), (101), (230) and (420) Bragg reflections. For the powdered Sb_2Se_3, the intensity ratios of (iii) and (221) XRD peaks (e.g. I_{120}/I_{221}= 0.55) are below 0.7 (Table 1). For the pristine and treated Sb_2Se3 films, all the intensity ratios of (iii) and (221) XRD peaks exceed 0.75. Thus, the crystallites in Sb_2Se_3 films are grown along several planes parallel to the substrate irrespective of the treatment performed.

Table S1. Ratios of intensities of (iii) and (221) diffraction peaks for the pristine and treated Sb_2Se_3 samples.

Ratio of intensities	Sb_2Se_3 powder PDF 01-083-1758	Sb_2Se_3 films				
		As-dep	50 mM	150 mM	300 mM	450 mM
$I_{(120)}/I_{(221)}$	0.55	5.66	3.61	1.42	5.23	5.13
$I_{(101)}/I_{(221)}$	0.12	2.73	1.70	0.75	2.77	2.65
$I_{(230)}/I_{(221)}$	0.70	6.18	3.42	1.52	5.23	5.05
$I_{(420)}/I_{(221)}$	0.20	4.82	3.15	1.26	4.68	4.3

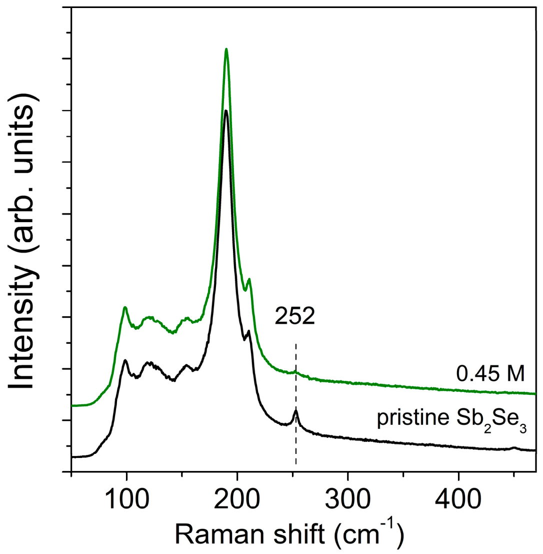

Figure S5. Raman spectra of the pristine Sb_2Se_3 film and those thermally treated in glycerol at 450 mM $SbCl_3$ for 90 min at 300 °C.

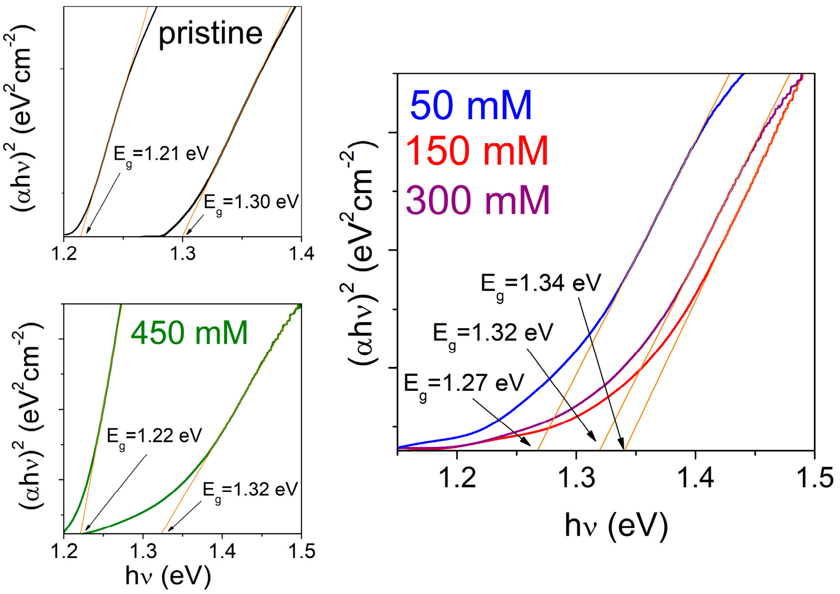

Figure S6. Tauc plots of the pristine Sb_2Se_3 film and those thermally treated in glycerol at various concentrations of $SbCl_3$ for 90 min at 300 °C. The direct band gap (E_g) values were found by extrapolating the straight-line portion of the $(\alpha h\nu)^2$ versus $h\nu$ graph to a zero-absorption coefficient value. The optical bandgap values are presented in Table 1.

Conversion between potentials vs. SCE and vs. RHE [1]

$$E \text{ (vs. } RHE) = E \text{ (vs. SCE)} + E^0 \text{ (SCE reference)} + 0.0591 \text{ V} \times \text{pH} \quad \text{(S1)}$$

$$E^0 \text{ (SCE reference)} = 0.248 \text{ V vs. NHE at 25 ºC}$$

The V_{OC} value visible in the LSV curves measured for the pristine Sb_2Se_3 film was -0.13 V vs. SCE, while the V_{OC} values of all treated Sb_2Se_3 films were - 0.05 V vs. SCE (Figure 4a).

$$E_{pristine} \text{ (vs. RHE)} = -0.13 \text{ V (vs. SCE)} + 0.248 \text{ (vs. NHE)} + 0.0591 \times 6.3 = 0.490 \text{ V} \quad \text{(S2)}$$

$$E_{treated} \text{ (vs. RHE)} = -0.05 \text{ V (vs. SCE)} + 0.248 \text{ (vs. NHE)} + 0.0591 \times 6.3 = 0.570 \text{ V} \quad \text{(S3)}$$

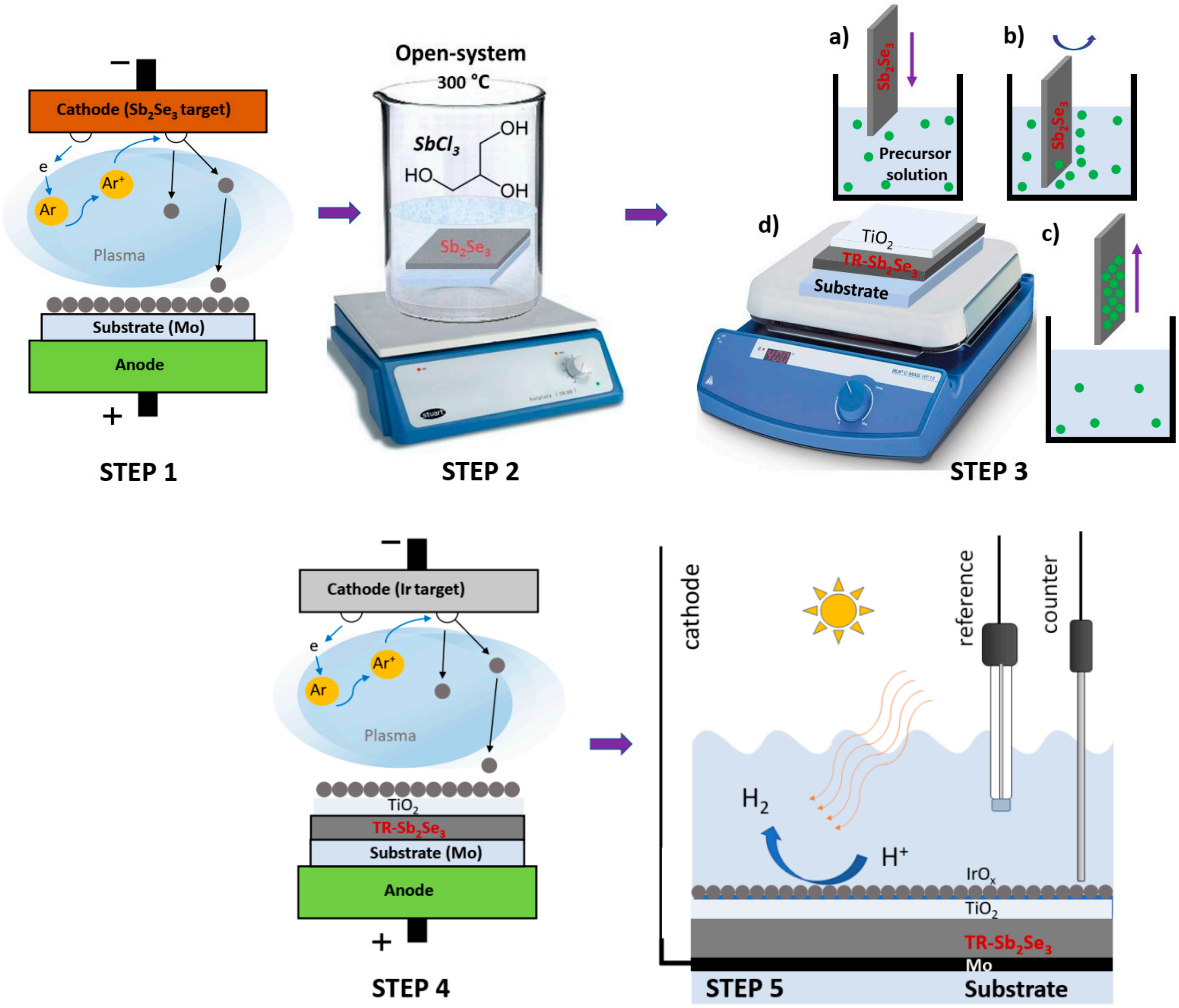

Figure S7. A sketch for the fabrication of Mo/treated-Sb_2Se_3/TiO_2/IrO_x structures along with an electrochemical cell under solar illumination.

Table S2. Neutral defects formation energies.

Position	Formation energy (eV)		
	Se-vacancy**	Sb-vacancy**	Cl**
1	2.0418	3.0897	0.3591
2	2.0719	3.0577	0.1861
3	2.0694	—	0.1895

** Se-, Sb- and Cl-rich condition

Theoretical calculation

Electronic structure of Sb_2Se_3 was studied by the Vienna ab initio simulation package (VASP) together with the potential projector augmented-wave (PAW) method [2–5]. Exchange and correlation effects were described by the Perdew-Burke-Ernzerhoff (PBE) of generalized gradient approximation as well as HSE06 hybrid functional containing a modified portion of the Fock exchange [6]. PAW-PBE pseudopotentials were employed to describe the Sb($4d^{10}5s^25p^3$) and Se($4s^24p^4$) valence states. Hybrid functional was performed as proposed by Heyd-Scuseria-Ernzerhof (HSE) [7,8]. In the hybrid functional a standard value of the (short-range) Hartree-Fock exchange (25%) is mixed with a portion of PBE exchange (75%). The screening parameter of 0.25 $Å^{-1}$ is used. Structural optimization was performed with k-mesh 8x8x8, plane-wave cut-off energy of 600 eV, and energy error of 10^{-8} eV. The residual forces and pressure are less than 10^{-4} eV/A and 0.06 kB, respectively.

References

1. Kim, T.W.; Choi, K.-S. Nanoporous BiVO4 Photoanodes with Dual-Layer Oxygen Evolution Catalysts for Solar Water Splitting. *Science* **2014**, *343*, 990–994, doi:10.1126/science.1246913.
2. Kresse, G.; Furthmüller, J. Efficient iterative schemes for ab initio total-energy calculations using a plane-wave basis set. *Phys. Rev. B*. **1996**, *54*, 11169–11186.
3. Kresse, G.; Furthmüller, J. Efficiency of ab-initio total energy calculations for metals and semiconductors using a plane-wave basis set. *Comput. Mater. Sci.* **1996**, *6*, 15–50, doi:10.1016/0927-0256(96)00008-0.
4. Kresse, G.; Joubert, D. From ultrasoft pseudopotentials to the projector augmented-wave method. *Phys. Rev. B* **1999**, *59*, 1758–1775, doi:10.1103/physrevb.59.1758.
5. Blochl, P.E. Projector augmented-wave method. *Phys. Rev. B.* **1994**, *50*, 17953-17979.
6. Perdew, J.P.; Burke, K.; Ernzerhof, M. Generalized Gradient Approximation Made Simple. *Phys. Rev. Lett.* **1996**, *77*, 3865–3868, doi:10.1103/physrevlett.77.3865.
7. Krukau, A.V.; Vydrov, O.A.; Izmaylov, A.F.; Scuseria, G.E. Influence of the exchange screening parameter on the per-formance of screened hybrid functionals. *J. Chem. Phys.* **2006**, *125*, 224106.
8. Heyd, J.; Scuseria, G.E.; Ernzerhof, M. Hybrid functionals based on a screened Coulomb potential. *J. Chem. Phys.* **2003**, *118*, 8207–8215.
